# Effect of Annexin A2 on prognosis and sensitivity to immune checkpoint plus tyrosine kinase inhibition in metastatic renal cell carcinoma

**DOI:** 10.1007/s12672-024-00934-0

**Published:** 2024-03-22

**Authors:** Jiajun Wang, Jinglai Lin, Jiahao Wang, Ying Wang, Yanjun Zhu, Xianglai Xu, Jianming Guo

**Affiliations:** 1 Department of Urology, Zhongshan Hospital, Fudan University, No.180 Fenglin Road, Shanghai, 200032 China; 2 Department of Urology, Zhongshan Hospital (Xiamen), Fudan University, Xiamen, 361015 China; 3 Xiamen Clinical Research Center for Cancer Therapy, Zhongshan Hospital (Xiamen), Fudan University, Xiamen, 361015 China; 4 Department of Critical Care Medicine, Zhongshan Hospital, Fudan University, Shanghai, 200032 China

**Keywords:** Annexin A2, Renal cell carcinoma, IO + TKI combination, Therapeutic resistance, Tumor microenvironment

## Abstract

**Background:**

Immunotherapy (IO) plus tyrosine kinase inhibitor (TKI) therapy is the first-line recommendation for advanced renal cell carcinoma (RCC), but no biomarker has been approved for it. Annexin A2 (ANXA2) can induce immune escape in tumors.

**Methods:**

Two independent cohorts of advanced RCC treated by IO + TKI were utilized for survival analysis (ZS-MRCC, n = 45; Javelin-101, n = 726). ANXA2 expression was determined by RNA-sequencing. The impact of ANXA2 on the tumor microenvironment was assessed by RNA-sequencing, flow cytometry and immunohistochemistry in two localized RCC datasets (ZS-HRRCC, n = 40; TCGA-KIRC, n = 530).

**Results:**

ANXA2 was upregulated in non-responders of IO + TKI therapy (p = 0.027). High-ANXA2 group showed poor progression-free survival (PFS) in both the ZS-MRCC cohort (HR, 2.348; 95% CI 1.084–5.085; P = 0.025) and the Javelin-101 cohort (HR, 1.472; 95% CI 1.043–2.077; P = 0.027). Multivariate Cox regression determined ANXA2 as an independent prognostic factor (HR, 2.619; 95% CI 1.194–5.746; P = 0.016). High-ANXA2 was correlated with decreased proportion of granzyme B^+^ CD8^+^ T cells (Spearman’s ρ = − 0.40, P = 0.01), and increased TIM-3^+^ (Spearman’s ρ = 0.43, P < 0.001) and CTLA4^+^ (Spearman’s ρ = 0.49, P < 0.001) tumor-infiltrating lymphocytes. A random forest (RF) score was further build by integrating ANXA2 and immune genes, which stratified patients who would benefit from IO + TKI therapy (low-RF score, IO + TKI vs TKI, HR = 0.453, 95% CI 0.328–0.626; high-RF score, IO + TKI vs TKI, HR = 0.877, 95% CI 0.661–1.165; interaction P = 0.003).

**Conclusions:**

Upregulated ANXA2 was associated with poor PFS and therapeutic resistance in RCC treated by IO + TKI therapy, and related with T cell exhaustion. The integrated RF score could stratify patients who would benefit from IO + TKI therapy.

**Supplementary Information:**

The online version contains supplementary material available at 10.1007/s12672-024-00934-0.

## Introduction

In 2020, more than 400,000 new cases of renal cell carcinoma (RCC) were reported worldwide, which accounts for approximately 3% of all adult cancers [1], and the majority of the cases were clear cell renal cell carcinomas (ccRCC) [2]. Over 30% of the RCC patients were diagnosed with metastases, with a 5-year survival rate of approximately 10% [3].

Recent randomized controlled trials have demonstrated the fundamental role of immunotherapy (IO) plus tyrosine kinase inhibitor (TKI) for metastatic RCC, with superior compared with TKI monotherapy [4–7]. Nonetheless, a significant proportion of patients continue to exhibit inherent resistance to IO + TKI or develop acquired resistance [6–8]. To aid clinical decision-making, novel biomarkers for IO + TKI therapy in RCC are urgently required.

Annexin A2 (ANXA2) is one of the members of the annexin family, which regulates cellular development and signal transduction pathways [9, 10]. ANXA2 has been reported to be upregulated in multiple cancer types, including RCC [9, 10]. ANXA2 has also been demonstrated to be involved in tumor angiogenesis [11] and metastasis [12]. In RCC, Annexin A2 was reported to be associated with higher tumor grade, metastatic potential and poor prognosis [13, 14]. Elevated ANXA2 expression was also able to promote RCC cell motility and migration [15]. Recently, ANXA2 was also found to regulate Hippo signaling pathway, and may be a potential target for pharmaceutical intervention in RCC [16]. However, the mechanisms underlying ANXA2 function in RCC remain obscure.

In recent years, increasingly more studies have focused on the relationship between ANXA2 and tumor microenvironment [17]. ANXA2 was found to be essential for T cell to T cell interactions, and the upregulation of ANXA2 leads to decreased T-cell activation in tumor microenvironment [18]. Annexin A2 also promotes immune escape of hepatocellular carcinoma, by upregulation of immune checkpoint molecules, and decreased expression of effector factors, including perforin, granzyme B (GZMB), interferon-γ, and TNF-α [17]. Moreover, soluble ANXA2 also showed immunosuppressive properties in RCC [19], and elevated serum levels of ANXA2 may be important for the suppression of the immune response [20]. These growing evidences showed the important role of ANXA2 in tumor immune escape. In fact, ANXA2 contributes to therapeutic resistance of immunotherapy [21]. Accordingly, preclinical studies have been evaluating the anti-tumor efficacy of Annexin A2-targeting therapy [22], alone or in combination with anti-PD-1 antibodies [23, 24]. However, these is no study evaluating the prognostic or predictive role of ANXA2 for IO + TKI therapy in RCC.

In the study, we aimed to evaluate the prognostic role of ANXA2 in advanced RCC treated by IO + TKI, in two independent cohorts. We intended to explore the function of ANXA2 in tumor microenvironment of RCC. We also aimed to build a predictive model for IO + TKI therapy benefit in advanced RCC.

## Materials and methods

### Study cohorts and datasets

This study included two advanced RCC cohorts, the Zhongshan Hospital metastatic renal cell carcinoma (ZS-MRCC) cohort, and the Javelin-101 cohort.

The inclusion criteria of the ZS-MRCC cohort were RCC metastasis, IO + TKI therapy, no other malignancies, and sample availability. Fifty-one patients were initially enrolled, and therapeutic responses were evaluated every 6–8 weeks after treatment initiation according to computed tomography. Therapeutic responses were defined as complete response (CR), partial response (PR), stable disease (SD) or progressive disease (PD) according to the Response Evaluation Criteria in Solid Tumors (RECIST) 1.1 criteria [25]. Patients of CR or PR were defined as responders, and those of SD or PD were non-responders. The primary endpoint of the study was PFS, defined as the time from IO + TKI therapy initiation to disease progression or death. Six patients were excluded due to unavailable sample, or loss of follow-up. Finally, the ZS-MRCC cohort included 45 patients. Baseline characteristics of the ZS-MRCC cohort are displayed in Supplementary Table S1. The correlations between baseline characteristics and therapeutic response are listed in Supplementary Table S2.

The Javelin-101 cohort included 726 patients from the phase III Javelin-101 trial of advanced RCC [7]. In this cohort, 354 patients received IO + TKI, while 372 patients received TKI monotherapy. The inclusion and exclusion criteria have been previously described [7]. Patients’ characteristics, genomic data, transcriptomic data and PFS were acquired from the previous studies [7, 26]. Baseline characteristics of the Javelin-101 cohort are listed in Supplementary Table S3.

This study also included another two datasets for transcriptomic, immunologic and functional studies, the Zhongshan Hospital high risk renal cell carcinoma (ZS-HRRCC) dataset, and the Cancer Genome Atlas Clear Cell Kidney Cancer (TCGA-KIRC) dataset. The ZS-HRRCC dataset initially enrolled 43 high-risk localized RCC patients. Three patients were excluded for sample unavailability or not passing sample quality control. The remaining 40 patients’ samples were used for analysis. The TCGA-KIRC dataset included 530 samples of clear cell RCC [27]. Patients’ characteristics, survival, genomic and transcriptomic data were all downloaded from the UCSC xena browser (https://xena.ucsc.edu/) [27].

The study was approved by the Clinical Research Ethics Committee of Zhongshan Hospital, Fudan University (B2021-119), following the Declaration of Helsinki. Written informed consent was obtained from each participant.

### RNA-sequencing procedures

RNA-sequencing were performed in the ZS-MRCC cohort and the ZS-HRRCC dataset. For total RNA isolation, the MagBeads Total RNA Extraction Kit (Catalog #T02-096) was used according to the manufacturer’s instructions. The RNAClean XP Kit (Cat#A63987, Beckman Coulter, Inc. Kraemer Boulevard Brea, CA, USA) and the RNase-Free DNase Set (Cat#79254, QIAGEN, GmBH, Germany) were used for total RNA purification. Library construction and sequencing was performed by Shanghai Biotechnology Corp. (Shanghai, China). For RNA library preparation and sequencing, VAHTS Universal V6 RNA-sequencing Library Prep Kit for Illumina (Cat#NR604-02, Vazyme, Nanjing, China) and NovaSeq 6000 equipment (Illumina, USA) were utilized. RNA-sequencing data were standardized to read count and Fragments Per Kilobase of exon model per Million mapped fragments (FPKM).

### Hematoxylin and eosin (H&E) staining and immunohistochemistry (IHC)

Hematoxylin and eosin (H&E) staining and immunohistochemistry were performed on formalin-fixed, paraffin-embedded RCC slides in the ZS-HRRCC dataset. Tumor-infiltrating lymphocytes (TILs) were assessed by H&E staining, according to previously described methodology by the International TILs Working Group [28]. IHC was performed according to previously described procedures [29]. IHC markers and primary antibodies are listed in Supplementary Table S4. The PANNORAMIC® 250 Flash III DX (3DHISTECH Ltd.) and the CaseViewer application (3DHISTECH Ltd.) were used for image capture and display. IHC analysis was conducted under six randomized fields for each sample, by three independent investigators blinded to patients’ information. The average value was calculated for further analysis.

### Flow cytometry

Peripheral blood samples were collected before surgery, stored in heparinized tubes at 4 °C within two hours before the experiment. RBC Lysis Buffer (Thermo Fisher Scientific) was used to isolate white blood cells. RCC samples were collected and minced after resection. After digestion by collagenase IV (Sigma) and DNase I (Sigma) at 37 °C and passing through a 70-μm strainer, RCC samples were then treated with RBC lysis buffer (Thermo Fisher Scientific) to obtain single cell suspensions. Samples were then treated with Fc receptors blockade, and stained for 30 min at 4 °C with fluorescently labeled membrane marker antibodies. Intracellular proteins were stained with corresponding antibodies after being disposed by Intracellular Fixation and Permeabilization Buffer (Thermo Fisher Scientific). Samples were then stained by the fluorochrome-labeled antibodies and preserved in cell staining buffer. Flow-cytometry analysis were performed by BD LSRFortessa™ X-20 (BD Biosciences) and analyzed by Flowjo v10.0 (Tree Star). Antibodies used for flow cytometry are listed in Supplementary Table S4.

### Random forest model construction

Random forest (RF) is a nonparametric approach for classification and regression [30], which is suitable for analyzing survival and omics data [31, 32]. Expression of ANXA2, PDCD1, CD4, GZMB, CD8A, CD274, CTLA4, GZMK were included as parameters for the construction of the random forest score (RF score). The random forest model was constructed by the “randomForestSRC” and “ggRandomForests” packages of the R software.

### Statistical analysis

Chi-square test, Fisher’s exact analysis or Cochran–Mantel–Haenszel test were used for categorical variables. Wilcoxon signed-rank test or Kruskal–Wallis H test were used for continuous variable. Spearman’s correlation test was used for correlation analysis. Kaplan–Meier analysis with log-rank test and Cox regression analysis were used for survival analysis, by the “survival” and “survminer” packages of the R software. The forest plots were plotted by the “forestplot” package, and the waterfall plot was plotted by the “ComplexHeatmap” and “ggplot2” packages of the R software. All statistical analyses were performed by using R software (https://www.r-project.org/). For all statistical analyses, two-tailed P < 0.05 was regarded as statistically significant.

## Results

### Upregulated ANXA2 expression correlated with RCC progression

**Fig. 1:**
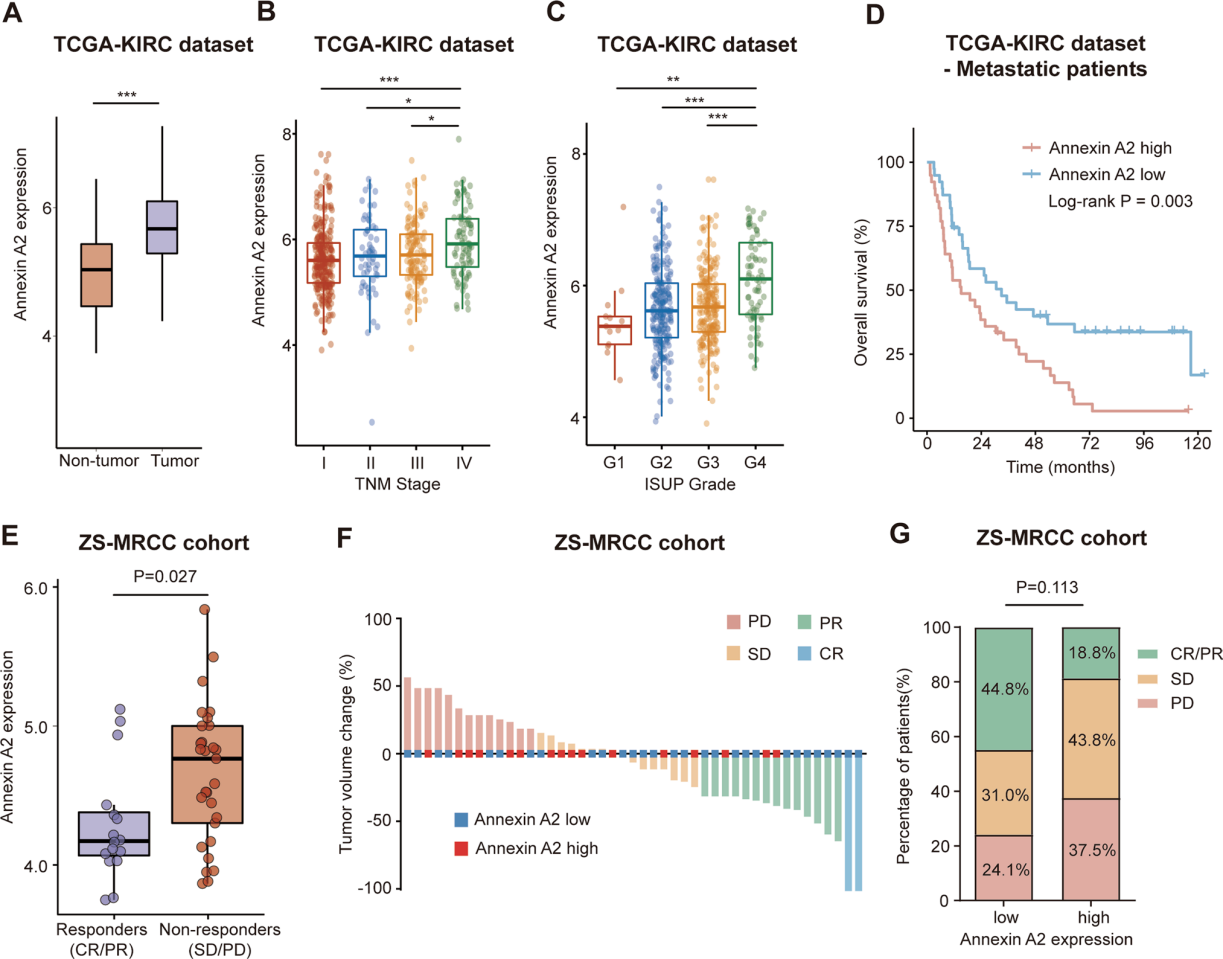
Elevated ANXA2 expression is associated with therapeutic resistance to immunotherapy (IO) plus tyrosine kinase inhibitor (TKI) in advanced RCC. **A** ANXA2 expression in tumor and peritumor tissues of RCC from the TCGA-KIRC dataset. P value, Mann–Whitney U-test. ***P < 0.001. **B**, **C** ANXA2 expression in tumor samples of different TNM stage (**B**) and ISUP grade (**C**) from the TCGA-KIRC dataset. P values, Kruskal–Wallis H test. *P < 0.05. **P < 0.01. ***P < 0.001. **D** Overall survival according to ANXA2 expression in metastatic RCC patients from the TCGA-KIRC dataset. P value, log-rank test. **E** Tumor ANXA2 expression between responders and non-responders of IO + TKI therapy from the ZS-MRCC cohort. Responders, complete response (CR) or partial response (PR). Non-responders, stable disease (SD) or progressive disease (PD). P value, Mann–Whitney U-test. **F** Best tumor volume change from baseline after IO + TKI therapy in the ZS-MRCC cohort. **G** RECIST response after IO + TKI therapy in ANXA2 high/low groups of the ZS-MRCC cohort. P value, Mann–Whitney U-test

Change of ANXA2 expression during RCC progression was evaluated in the TCGA-KIRC dataset. ANXA2 expression was upregulated in RCC samples, compared with non-tumor kidney tissues (P < 0.001, Fig. 1A). ANXA2 expression was also upregulated in TNM stage IV RCC (stage IV vs stage I, P < 0.001; stage IV vs stage II, P < 0.05; stage IV vs stage III, P < 0.05; Fig. 1B), as well as ISUP grade 4 tumors (grade 4 vs grade 1, P < 0.01; grade 4 vs grade 2, P < 0.001; grade 4 vs grade 3, P < 0.001; Fig. 1C). In addition, upregulated ANXA2 expression correlated with poor prognosis in metastatic RCC patients (P = 0.003, Fig. 1D).

### Upregulated ANXA2 expression correlated with resistance to IO + TKI therapy

ANXA2 expression was evaluated by RNA-sequencing in the ZS-MRCC cohort. Upregulated ANXA2 expression was found in non-responders under IO + TKI therapy, compared with responders (P = 0.027, Fig. 1E). Patients with low-ANXA2 expression were more likely to respond to IO + TKI therapy, while those with high ANXA2 expression were not, although not statistically significant (P = 0.113, Fig. 1F, G).

### High-ANXA2 correlated with poor PFS under IO + TKI therapy

**Fig. 2:**
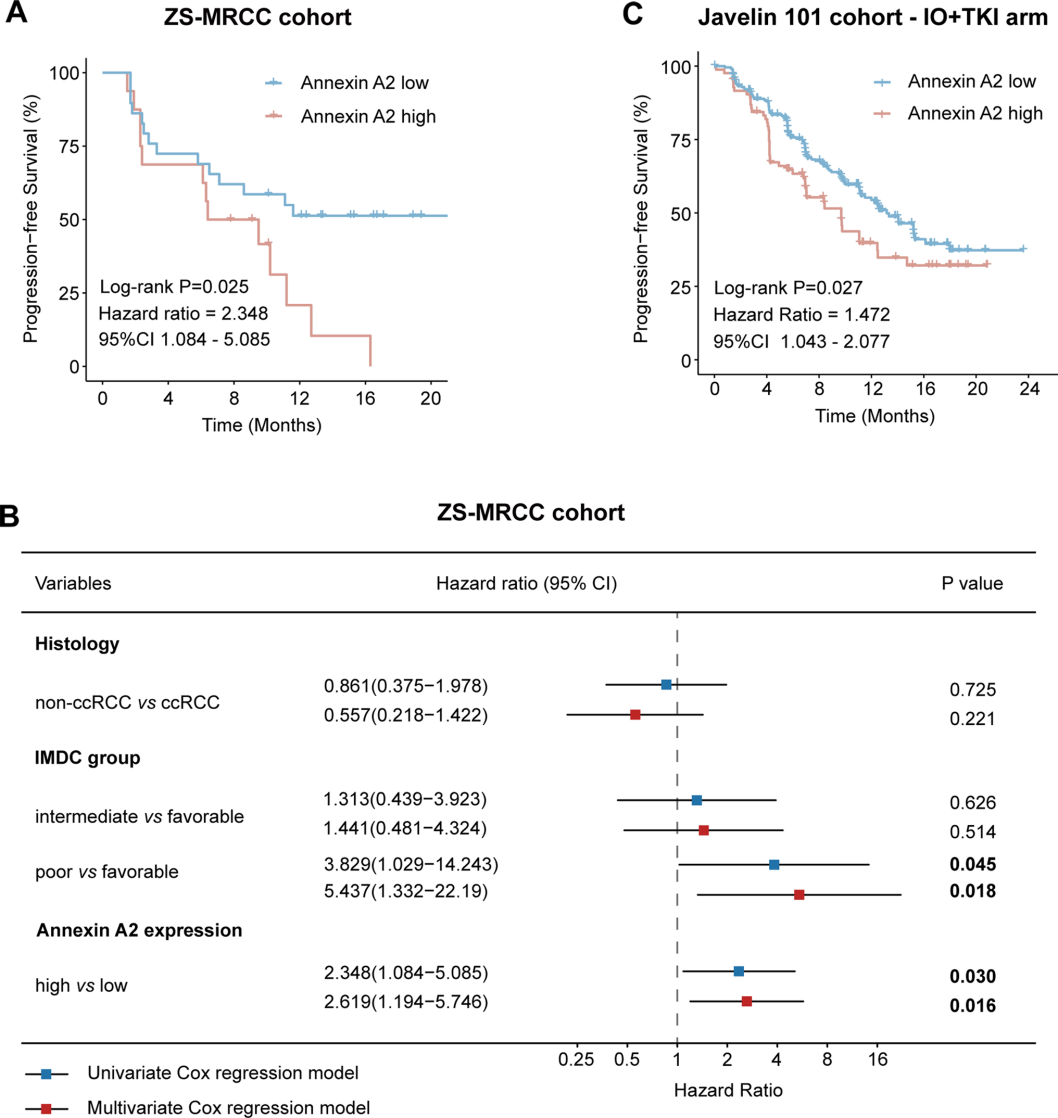
ANXA2 expression is related with poor progression-free survival (PFS) in advanced RCC treated by IO + TKI therapy. **A** PFS according to ANXA2 expression in the IO-TKI treated patients from the ZS-MRCC cohort. P value, log-rank test. Hazard ratio, univariate Cox regression model. **B** Forest plot for univariate and multivariate Cox regression model for PFS, including histology, IMDC group, and ANXA2 expression. Hazard ratios and P values, Cox regression. **C** PFS according to ANXA2 expression in the IO-TKI treated patients from the Javelin-101 cohort. P value, log-rank test. Hazard ratio, univariate Cox regression model

The correlation between ANXA2 expression and PFS was evaluated in IO + TKI treated patients from the ZS-MRCC cohort and the Javelin-101 cohort. In the ZS-MRCC cohort, the high-ANXA2 group showed poor PFS than the low-ANXA2 group (log-rank P = 0.025; HR for univariate Cox regression, 2.348, 95 CI 1.084–5.085; Fig. 2A). In a multivariate Cox regression model including tumor histology, IMDC group and ANXA2 expression. ANXA2 was determined as an independent prognostic factor for poor PFS (HR, 2.619, 95 CI 1.194–5.746; P = 0.016, Fig. 2B). Moreover, high ANXA2 expression also indicated poor PFS in the ccRCC subgroup (P = 0.026, Supplementary Figure S1A) and the first-line therapy subgroup (P < 0.001, Supplementary Figure S1B). In the IO + TKI arm of the Javelin-101 cohort, high-ANXA2 group also displayed poor PFS, compared with low-ANXA2 group (log-rank P = 0.027; HR for univariate Cox regression, 1.472, 95 CI 1.043–2.077; Fig. 2C).

### Correlation between ANXA2 and tumor-infiltrating T cells

**Fig. 3:**
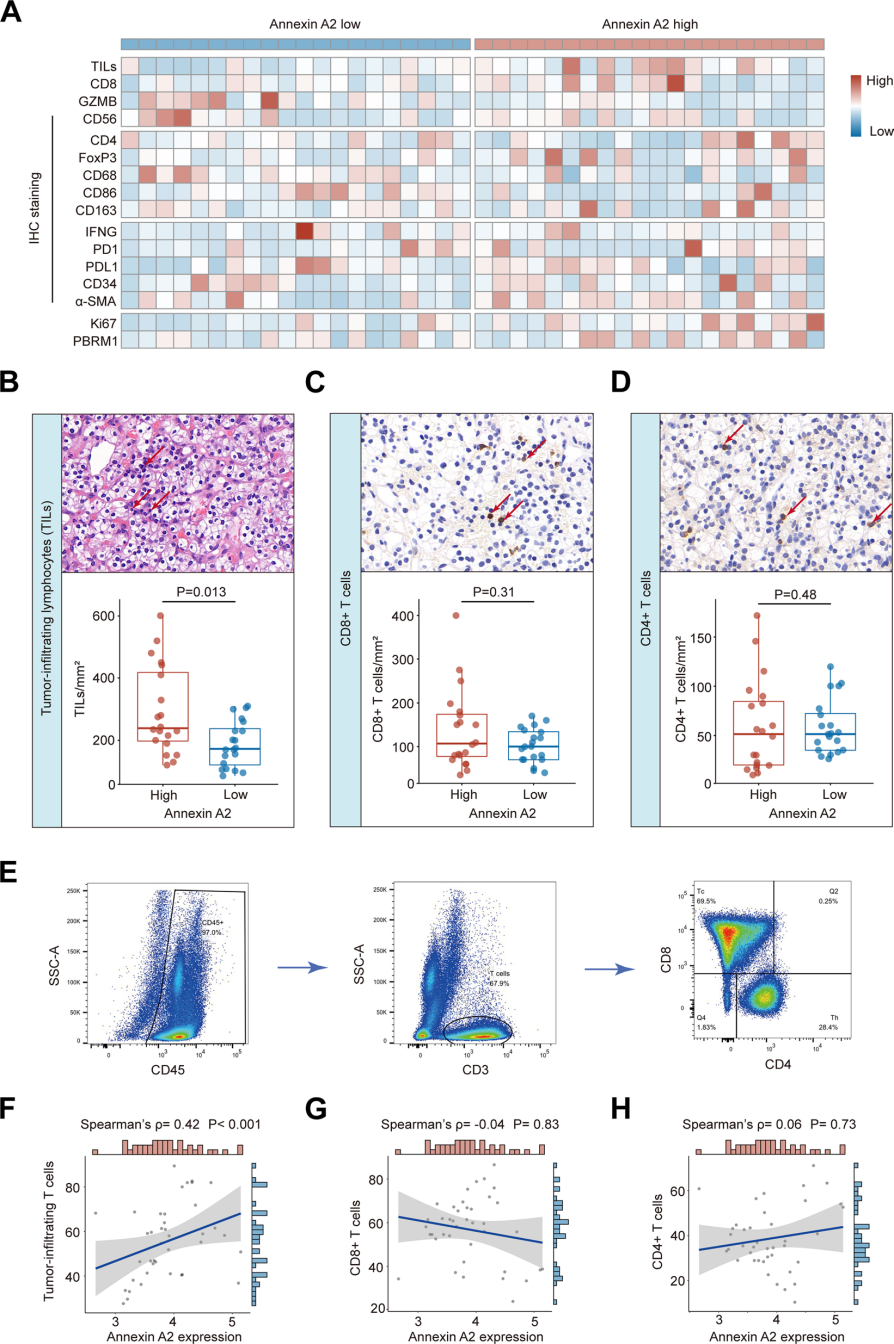
Relationship between ANXA2 expression and immune cell infiltration. **A** Heatmap of immunohistochemistry markers of tumor microenvironment in 40 localized RCC samples. Samples were ranked according to ANXA2 expression. **B**–**D** Representative images and quantification of tumor-infiltrating lymphocytes (**B**), CD8^+^ T cells (**C**), and CD4^+^ T cells (**D**) sorted by ANXA2 expression level. P values, Mann–Whitney U-test. **E** Representative images of gating strategy for tumor-infiltrating T cells, CD8^+^ T cells, and CD4^+^ T cells by flow cytometry. **F**–**H** Association between ANXA2 expression and tumor-infiltrating T cells (**F**), CD8^+^ T cells (**G**), and CD4^+^ T cells (**H**) by flow cytometry. ρ and P values, Spearman’s correlation

The relationship between ANXA2 and tumor-infiltrating immune cells was evaluated by H&E or IHC in RCC (Fig. 3A). TILs were elevated in high-ANXA2 samples, compared with low-ANXA2 samples (P = 0.013, Fig. 3B). However, the number of CD8^+^ T cells or CD4^+^ T cells did not show difference between high/low ANXA2 samples (P = 0.31, Fig. 3C; P = 0.48, Fig. 3D). Further validation was performed by flow cytometry (Fig. 3E). Similarly, ANXA2 expression displayed positive correlation with tumor-infiltrating T cells (Spearman’s ρ = 0.42, P < 0.001, Fig. 3F), while it did not show significant correlation with CD8^+^ T cells (P = 0.83, Fig. 3G) or CD4^+^ T cells (P = 0.73, Fig. 3H).

### Upregulated ANXA2 correlated with T cell exhaustion within RCC

**Fig. 4:**
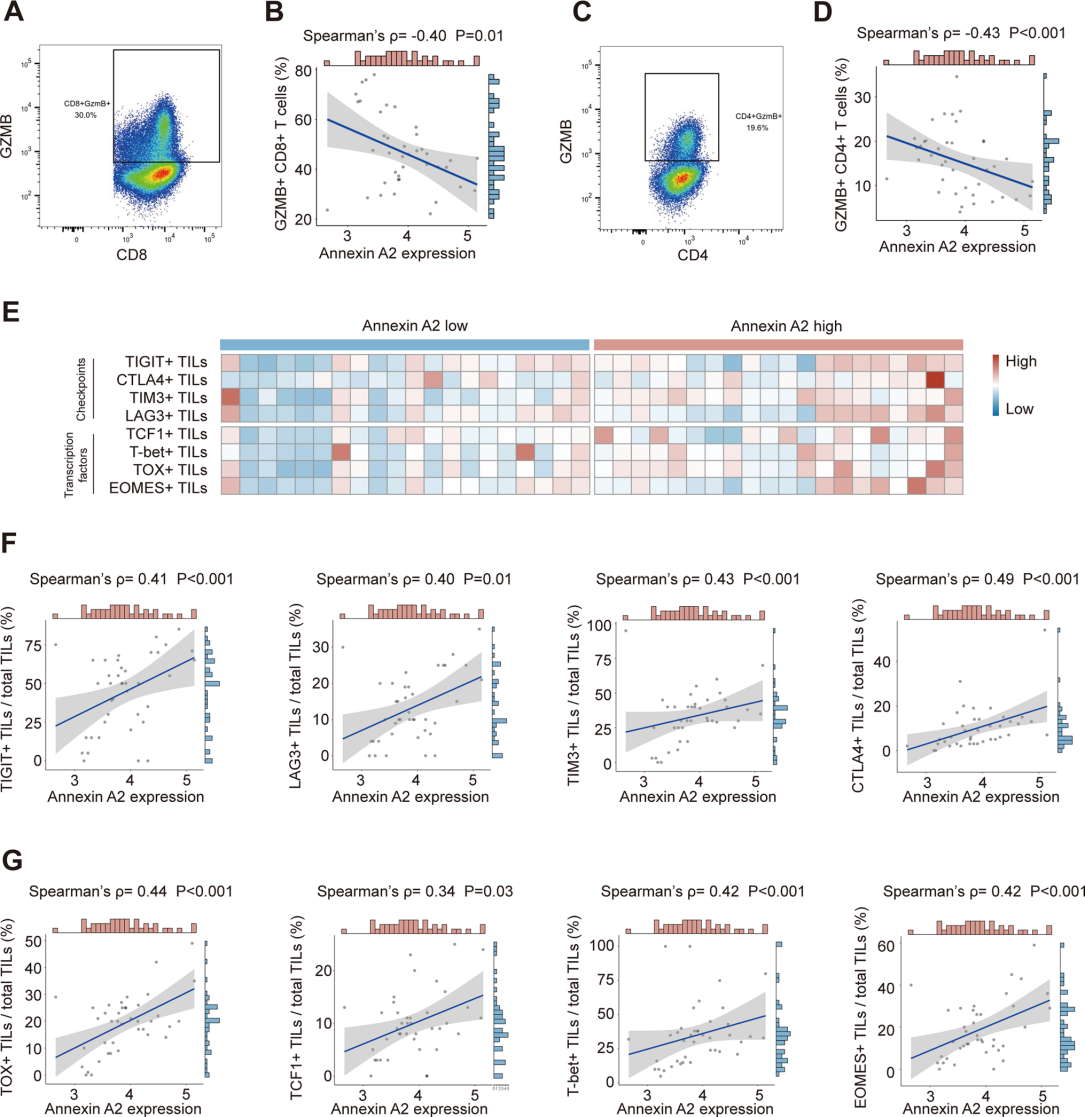
ANXA2 expression associated with T cell exhaustion in RCC tumor microenvironment. **A** Representative image of gating strategy for GZMB^+^ CD8^+^ T cells by flow cytometry. **B** Association between ANXA2 expression and GZMB^+^ CD8^+^ T cells. ρ and P value, Spearman’s correlation. **C** Representative image of gating strategy for GZMB^+^ CD4^+^ T cells by flow cytometry. **D** Association between ANXA2 expression and GZMB^+^ CD4^+^ T cells. ρ and P value, Spearman’s correlation. **E** Heatmap of T cell exhaustion markers expression in RCC microenvironment. Samples were ranked according to ANXA2 expression. **F** Association between ANXA2 expression and TIGIT^+^ T cells, LAG3^+^ T cells, TIM3^+^ T cells, and CTLA4^+^ T cells. ρ and P values, Spearman’s correlation. **G** Association between ANXA2 expression and TOX^+^ T cells, TCF1^+^ T cells, T-bet^+^ T cells, and EOMES^+^ T cells. ρ and P values, Spearman’s correlation

Flow cytometry was further performed to determine the function of T cells within RCC. ANXA2 expression was negatively correlated with the percentage of GZMB^+^CD8^+^ T cells (Spearman’s ρ = − 0.40, P = 0.01, Fig. 4A, B) as well as GZMB^+^CD4^+^ T cells (Spearman’s ρ = − 0.43, P < 0.001, Fig. 4C, D).

The expression of checkpoint molecules and transcription factors of T cells was further assessed by IHC (Fig. 4E). ANXA2 displayed positive correlation with checkpoint molecules, including TIGIT (Spearman’s ρ = 0.41, P < 0.001), LAG3 (Spearman’s ρ = 0.40, P = 0.01), TIM3 (Spearman’s ρ = 0.43, P < 0.001) and CTLA4 (Spearman’s ρ = 0.49, P < 0.001) (Fig. 4F). In addition, ANXA2 also showed positive correlation with transcription factors for T cell exhaustion, including TOX (Spearman’s ρ = 0.44, P < 0.001), TCF1 (Spearman’s ρ = 0.34, P = 0.03), T-bet (Spearman’s ρ = 0.42, P < 0.001) and EOMES (Spearman’s ρ = 0.42, P < 0.001) (Fig. 4G).

### Correlation between ANXA2 and suppressive factors within TME

**Fig. 5:**
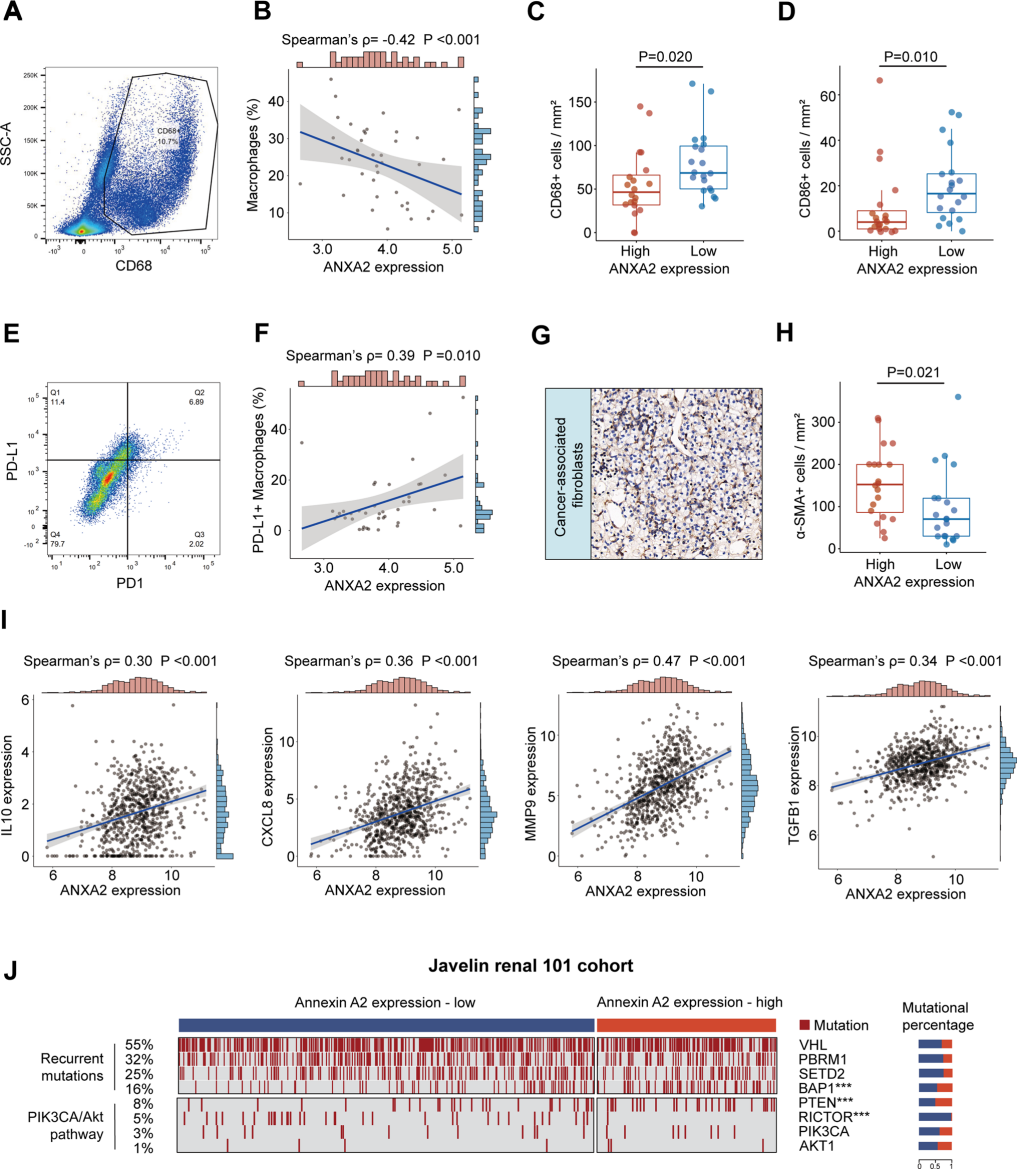
ANXA2 expression associated with macrophage phenotype in RCC tumor microenvironment. **A** Representative image of gating strategy for macrophages by flow cytometry. **B** Association between ANXA2 expression and macrophages. ρ and P value, Spearman’s correlation. **C**, **D** Quantification of CD68^+^ macrophages (**C**) and CD86^+^ M1-macrophages (**D**) by immunohistochemistry in high-ANXA2 and low-ANXA2 samples. **E** Representative image of gating strategy for PDL1^+^ macrophages by flow cytometry. **F** Association between ANXA2 expression and PDL1^+^ macrophages. ρ and P value, Spearman’s correlation. **G**, **H** Representative image and quantification of cancer-associated fibroblasts sorted by ANXA2 expression level. P value, Mann–Whitney U-test. **I** Correlation between ANXA2 expression and immunosuppressive cytokines. ρ and P values, Spearman’s correlation. **J** Waterfall plot of mutations according to ANXA2 expression. P values, Chi-square test. ***P < 0.001

Other immune cells apart from lymphocytes were also evaluated by flow cytometry and IHC in RCC in the study. ANXA2 showed negative correlation with macrophages, assessed by flow cytometry (Spearman’s ρ = − 0.42, Fig. 5A, B), and this correlation was also determined by IHC (P = 0.020, Fig. 5C). Interestingly, tumor-infiltrating M1-macrophages were also decreased in high-ANXA2 samples (P = 0.010, Fig. 5D). Moreover, ANXA2 expression was positively correlated with PDL1^+^ macrophages (Spearman’s ρ = 0.39, P = 0.010, Fig. 5E, F). Besides, cancer-associated fibroblasts were also more abundant in high-ANXA2 samples as well (P = 0.021, Fig. 5G, H). In addition, ANXA2 was positively correlated with suppressive factors, including IL10 (Spearman’s ρ = 0.30, P < 0.001), CXCL8 (Spearman’s ρ = 0.36, P < 0.001), MMP9 (Spearman’s ρ = 0.47, P < 0.001) AND TGF-β (Spearman’s ρ = 0.34, P < 0.001) (Fig. 5I).

### Correlation between ANXA2 and RCC mutations

Correlation between ANXA2 and tumor-driving mutations was assessed in the Javelin-101 cohort (Fig. 5J). High-ANXA2 samples had a higher incidence of BAP1 mutations (P < 0.001, Fig. 5J) and PTEN mutations (P < 0.001, Fig. 5J). However, high-ANXA2 samples displayed a lower incidence of RICTOR mutations (P < 0.001, Fig. 5J).

### An integrated model for predicting IO + TKI therapeutic benefit

**Fig. 6:**
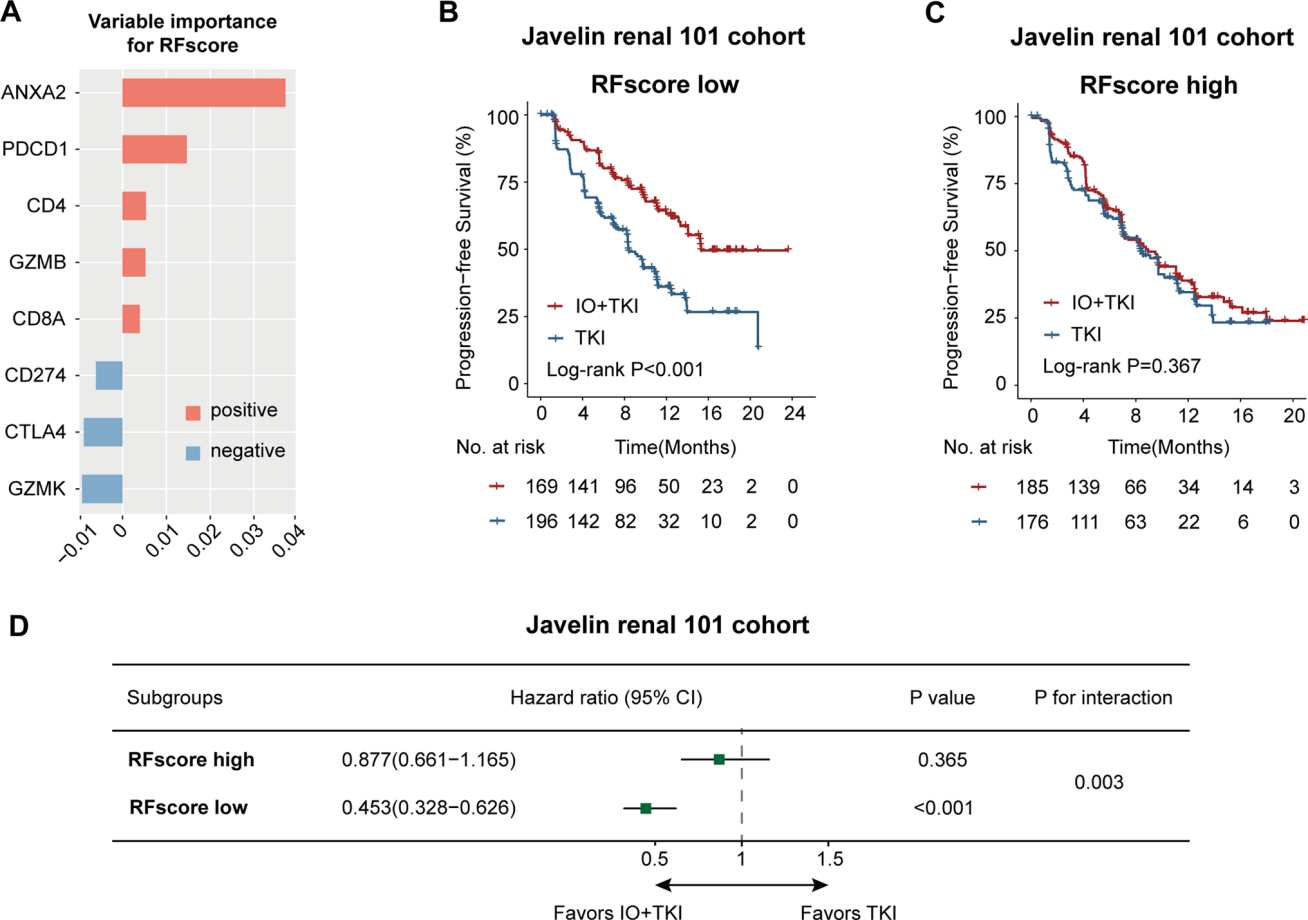
An integrated risk score for IO + TKI benefit prediction. **A** Genes enrolled for building random forest score (RF score). Genes ranked by variable importance. **B**, **C** PFS of IO + TKI combination or TKI monotherapy in the low RF score group (**B**) and high RF score group (**C**) in the Javelin renal 101 cohort. P values, log-rank test. **D** Forest plot of survival benefit between IO + TKI and TKI in the low/high RF score groups from the Javelin renal 101 cohort. Hazard ratios and P values, Cox regression test. P for interaction, Cox regression test

An integrated model for predicting IO + TKI therapeutic benefit was further constructed by random forest classification algorithm. This random forest score (RF score) included eight genes: ANXA2, PDCD1, CD4, GZMB, CD8A, CD274, CTLA4, GZMK (Fig. 6A). In the Javelin 101 cohort, IO + TKI therapy displayed better survival, compared with TKI monotherapy, only in the low-RF score group (log-rank P < 0.001, Cox regression HR = 0.453, 95% CI 0.328–0.626, Fig. 6B, D), but it showed no benefit in the high-RF score group (log-rank P = 0.367, Cox regression HR = 0.877, 95% CI 0.661–1.165, Fig. 6C, D). Therapeutic benefit of IO + TKI showed significant interaction with RF score subgroups, by interaction test (P for interaction = 0.003, Fig. 6D).

## Discussion

The combination therapy of IO + TKI has greatly improved the survival of advanced RCC patients [4–7], but there is still no available biomarker for it. ANXA2 has been found to take part in tumor immune escape [17–20], and therapeutic resistance of immunotherapy [21]. Accordingly, preclinical studies have been evaluating the anti-tumor efficacy of ANXA2-targeting therapy [22–24]. In the current study, high-ANXA2 indicated resistance and poor PFS of IO + TKI therapy, in two independent RCC cohorts. High-ANXA2 was also related with T cell dysfunction, and terminal exhaustion factors. High-ANXA2, combined with immune parameters, could stratify patients who would benefit from IO + TKI therapy.

Although IO + TKI has been proved to be superior than TKI monotherapy by phase III trials, there are still a great proportion of patients that could not benefit from it [4–7]. However, no biomarker has been applied yet. Biomarker-guided therapy selection between IO and TKI has shown potential benefit [33], but no biomarker is currently available for the selection between IO + TKI and TKI. In the current study, the low ANXA2 group showed a higher rate of tumor shrinkage (CR or PR, 44.8%) compared with the high ANXA2 group (CR or PR, 18.8%), and a lower rate of therapeutic resistance (PD, 24.1%) compared with the high ANXA2 group (PD, 37.5%), although not reaching statistical significance (Fig. 1G). To better represent therapeutic effect of IO/TKI, PFS was also calculated and set as the primary endpoint. ANXA2 correlated significantly with PFS in both the ZS-MRCC cohort and the Javelin-101 cohort (Fig. 2), demonstrating its prognostic value. The results also supported further investigations of ANXA2’s function in tumor immunity. A multivariate Cox regression model was also built to confirm the prognostic role of ANXA2 (Fig. 2B). The multivariate model included three risk factors: tumor histologic subtype (non-ccRCC or ccRCC), IMDC risk group (favorable, intermediate or poor) and ANXA2 expression (high or low). Among these, tumor histologic subtype and IMDC risk group are well-recognized prognostic factors for metastatic RCC. More importantly, the IMDC risk group is a widely-used risk stratification model for metastatic RCC, which includes six important prognostic factors: < 1 year from time of diagnosis to systemic therapy, Karnofsky performance status < 80%, hemoglobin < lower limit of normal, corrected calcium > upper limit of normal, neutrophils > upper limit of normal, and platelets > upper limit of normal. Accordingly, the multivariate model included most prognostic factors of metastatic RCC, which demonstrated ANXA2 expression as an independent factor for progression-free survival (Fig. 2B). More importantly, the integrated RF score could guide treatment selection between IO + TKI and TKI, as only low-RF score patients could benefit from the combined IO + TKI therapy (Fig. 6B–D). However, the potential application of ANXA2 and RF score still need further external validations. Additionally, since IO + IO has also been recommended for first-line therapy of metastatic RCC, further investigations are also expected for the role of ANXA2 in IO + IO therapy.

Previous studies reported up-regulated ANXA2 expression during RCC progression [13, 14]. In the current study, ANXA2 expression was also found upregulated in tumor tissues, especially in high-grade or high-stage RCC (Fig. 1A–C). As one of the members of the annexin family, ANXA2 is involved in multiple oncogenic pathways, including cellular development and signal transduction pathways [9, 10], angiogenesis [11], and metastasis [12]. In RCC, ANXA2 could regulate Hippo signaling pathway [16], and promote migration of tumor cells [15]. However, the mechanisms underlying ANXA2 function in RCC still remain obscure.

Previous evidences have indicated ANXA2 to be correlated with suppressive tumor microenvironment [17], especially correlated with decreased T-cell activation [18]. In the current study, ANXA2 was also found associated with T cell dysfunction, represented by decreased GZMB expression (Fig. 4A–D). In addition, ANXA2 was found correlated with exhaustion markers of T cells, including checkpoints (TIGIT, LAG3, TIM-3, CTLA4) and transcription factors (TOX, TCF1, T-bet, EOMES) (Fig. 4F, G). In hepatocellular carcinoma, ANXA2 has been reported to promotes immune escape, by upregulation of immune checkpoint molecules, and decreased expression of effector factors [17]. These evidences support the potential relevance between ANXA2 and immune suppression, but the mechanism has not clarified yet.

Soluble ANXA2 also showed immunosuppressive properties in RCC [19]. In our study, high-ANXA2 was related with increased number of PD-L1 + macrophages (Fig. 5E, F), and upregulated expression of suppressive factor, including IL10, CXCL8, MMP9 and TGF-β (Fig. 5I). The infiltration of suppressive macrophages, and overexpression of suppressive factors, are potential mechanism for immune suppression in high-ANXA2 samples. The immunosuppressive properties of ANXA2 may explain why high-ANXA2 tumors were resistance to IO + TKI. Accordingly, ANXA2 contributes to therapeutic resistance of immunotherapy [21], and ANXA2-targeting therapy in combination with anti-PD-1 antibodies has been evaluated in preclinical studies [23, 24]. However, the detailed mechanisms by which ANXA2 regulates T cell exhaustion and immunotherapy resistance need further researches.

The study has several limitations. Firstly, the retrospective design is the major limitation, leading to potential recall and enrollment biases. Secondly, the ZS-MRCC cohort enrolled patients of different histological subtypes. Although multivariate Cox analysis and hierarchical analysis were performed to avoid potential biases, the various histological subtypes may still become an important confounding factor. Besides, the ZS-MRCC cohort also enrolled patients of second-line or third-line IO + TKI therapy, mainly due to the fact that this regimen has only recently been approved for first-line treatment. In addition, the significantly different number of participants in the cohorts may lead to different results. However, despite the limited number of patients in the ZS-MRCC cohort, ANXA2 was still related with poor PFS, with statistical significance. Nevertheless, future validations in prospective trials will be more ideal.

## Conclusions

Upregulated ANXA2 expression could be an indicator for resistance and poor PFS in RCC treated by IO + TKI therapy. Upregulated ANXA2 expression was related with T cell dysfunction, and expression of exhaustion markers. The integrated RF score could stratify patients who could benefit from IO + TKI therapy.

### Supplementary Information

Below is the link to the electronic supplementary material.

Supplementary file 1 (TIF 825 KB)

Supplementary file 2 (DOC 49 KB)

Supplementary file 3 (DOC 53 KB)

Supplementary file 4 (DOC 38 KB)

Supplementary file 5 (DOC 54 KB)

## Data Availability

Data of the study can be shared to other researches upon reasonable request to Dr. Xianglai Xu (xl.xu@hotmail.com), according to data sharing policy.

## Code availability

Code of the study can be shared to other researches upon reasonable request to Dr. Xianglai Xu (xl.xu@hotmail.com).
