# Supplementary figures and images for: Effect of Annexin A2 on prognosis and sensitivity to immune checkpoint plus tyrosine kinase inhibition in metastatic renal cell carcinoma
Source: Discov Oncol. 2024 Mar 22;15:86. doi: 10.1007/s12672-024-00934-0 (PMC10959890; doi:10.1007/s12672-024-00934-0)

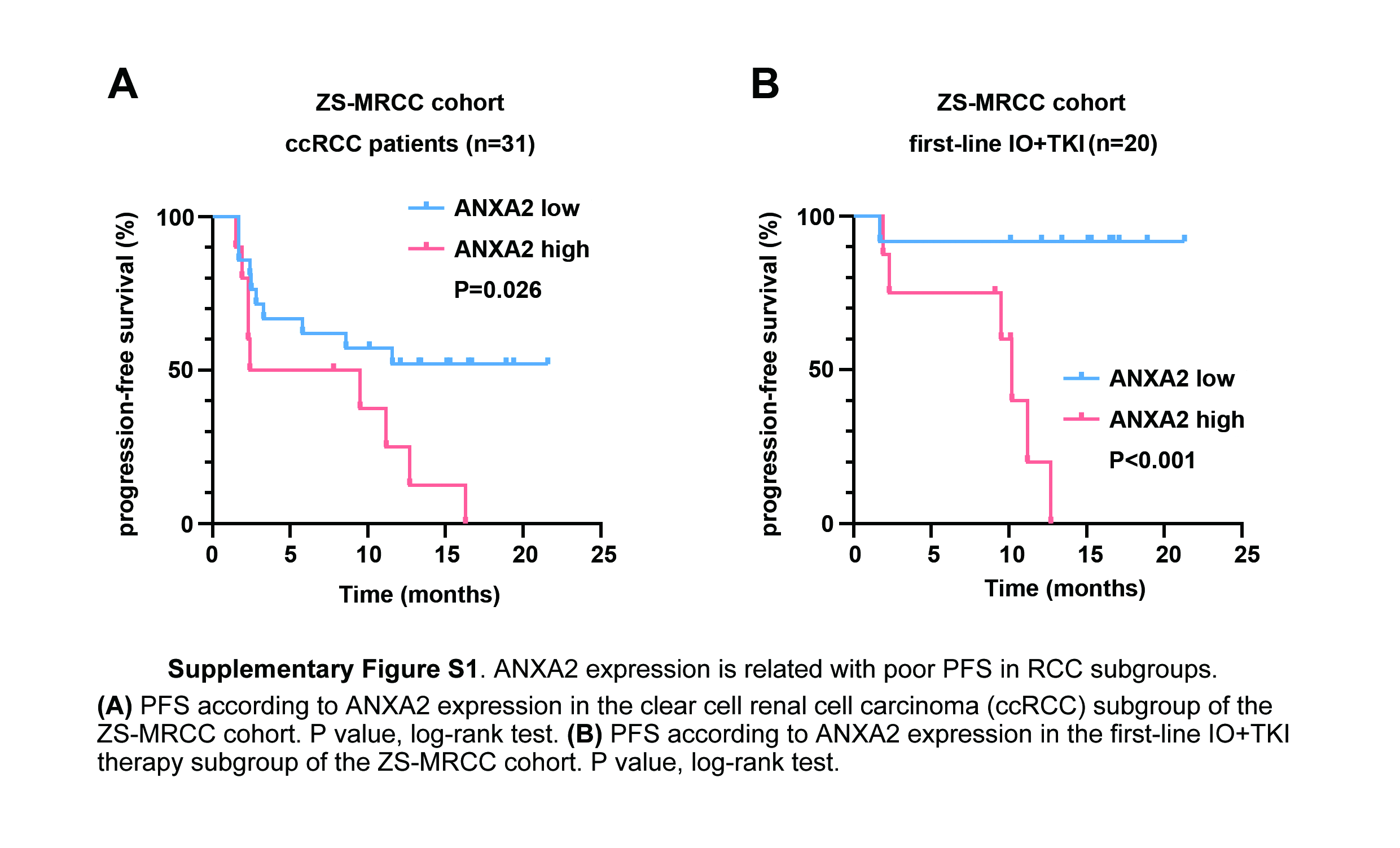

Supplement: Supplementary file 1 — (TIF 825 KB) [file 12672_2024_934_MOESM1_ESM.tif]
