# Supplementary material for: Effect of Annexin A2 on prognosis and sensitivity to immune checkpoint plus tyrosine kinase inhibition in metastatic renal cell carcinoma
Source: Discov Oncol. 2024 Mar 22;15:86. doi: 10.1007/s12672-024-00934-0 (PMC10959890; doi:10.1007/s12672-024-00934-0)
Supplement: Supplementary file 3 — (DOC 53 KB) [file 12672_2024_934_MOESM3_ESM.doc]

| Supplementary Table S2. Demographic and clinical characteristics according to therapeutic response in the ZS-MRCC cohort. | | | |
| --- | --- | --- | --- |
|  | Responders (CR/PR)  (n=16) | Non-responders (SD/PD)  (n=29) | P value |
| Age, median (range) | 59.5 (36-78) | 63 (18-79) | 0.731‡ |
| Gender |  |  | 0.486^§^ |
| Male | 10 (62.5%) | 15 (51.7%) |  |
| Female | 6 (37.5%) | 14 (48.3%) |  |
| Histology† |  |  | 0.228^§§^ |
| Clear cell | 13 (81.3%) | 18 (62.1%) |  |
| Papillary | 2 (12.5%) | 3 (10.3%) |  |
| Chromophobe | 0 (0%) | 1 (3.4%) |  |
| Xp11.2 Translocation | 0 (0%) | 3 (10.3%) |  |
| Sarcomatoid variants | 0 (0%) | 3 (10.3%) |  |
| Unclassified | 1 (6.3%) | 1 (3.4%) |  |
| ISUP grade |  |  | 0.531^§§§^ |
| II | 7 (46.7%) | 15 (53.6%) |  |
| III | 8 (53.3%) | 7 (25.0%) |  |
| IV | 0 (0%) | 6 (21.4%) |  |
| Regimens |  |  | 0.974^§§^ |
| Axitinib/Tislelizumab | 8 (50.0%) | 14 (48.3%) |  |
| Axitinib/Sintilimab | 3 (18.8%) | 5 (17.2%) |  |
| Lenvatinib/Pembrolizumab | 5 (31.3%) | 10 (34.5%) |  |
| Line of therapy |  |  | 0.238^§§§^ |
| First-line | 10 (62.5%) | 10 (34.5%) |  |
| Second-line | 2 (12.5%) | 11 (37.9%) |  |
| Third-line | 4 (25.0%) | 8 (27.6%) |  |
| IMDC risk group |  |  | 0.475^§§§^ |
| Favorable | 3 (18.8%) | 5 (17.2%) |  |
| Intermediate | 12 (75.0%) | 19 (65.5%) |  |
| Poor | 1 (6.3%) | 5 (17.2%) |  |
| ANXA2 expression |  |  | 0.080^§^ |
| High | 3 (18.8%) | 13 (44.8%) |  |
| Low | 13 (81.3%) | 16 (55.2%) |  |
| † Two patients were not classified for ISUP grade because of unclassified histology.  ‡ Kruskal–Wallis test by ranks.  ^§^ Chi-square test  ^§§^ Fisher’s exact test  ^§§§^ Cochran-Mantel-Haenszel test | | | |
