# Supplementary material for: Effect of Annexin A2 on prognosis and sensitivity to immune checkpoint plus tyrosine kinase inhibition in metastatic renal cell carcinoma
Source: Discov Oncol. 2024 Mar 22;15:86. doi: 10.1007/s12672-024-00934-0 (PMC10959890; doi:10.1007/s12672-024-00934-0)
Supplement: Supplementary file 4 — (DOC 38 KB) [file 12672_2024_934_MOESM4_ESM.doc]

| Supplementary Table S3. Baseline characteristics of the Javelin-101 cohort. | | | |
| --- | --- | --- | --- |
|  | All patients  n=726 | IO/TKI arm  n=354 | TKI arm  n=372 |
| Age, median (range) | 61 (27-88) | 62.5 (29-83) | 61 (27-88) |
| Gender |  |  |  |
| Male | 548 (75.5%) | 257 (72.6%) | 81 (21.8%) |
| Female | 178 (24.5%) | 97 (27.4%) | 271 (78.2%) |
| Regimen |  |  |  |
| Avelumab+Axitinib | 354 (48.8%) | 354 (100%) | 0 (0%) |
| Sunitinib | 372 (51.2%) | 0 (0%) | 372 (100%) |
| Histology |  |  |  |
| Clear cell | 726 (100%) | 354 (100%) | 372 (100%) |
| PD-L1 IHC |  |  |  |
| Negative | 210 (28.9%) | 109 (30.8%) | 101 (27.2%) |
| Positive | 516 (71.1%) | 245 (69.2%) | 271 (72.8%) |
